# Cognitive effects of partial and whole-body exposures to ^16^O particles

**DOI:** 10.1093/jrr/rrt187

**Published:** 2014-03

**Authors:** Bernard M. Rabin, Barbara Shukitt-Hale, Stacey Gomes, Kirsty L. Carrihill-Knoll

**Affiliations:** 1 Department of Psychology, UMBC, 1000 Hilltop Circle, Baltimore, MD 21250, USA; 2 Human Nutrition Research Center on Aging, USDA-ARS, Boston, MA 02111, USA

**Keywords:** behavior, cognition, HZE particles, space radiation

## Abstract

**Introduction:** When rats and mice are exposed to HZE particles at the NASA Space Radiation Laboratory (NSRL) at Brookhaven National Laboratory to simulate the effect of exposure to space radiation on cognitive performance, there may be differences in the amount of tissue that is irradiated: some experimenters irradiate only the head, whereas others irradiate the entire organism. Whether or not these different patterns of exposure have differential effects on cognitive performance remain to be established. Similarly, it remains to be established whether or not exposures restricted to the body can have an independent effect on cognitive performance.

**Radiation:** Rats were exposed to ^16^O (1000 MeV/n; 1, 5, 10, 25 cGy) particles at the NSRL. There were three exposure conditions: head-only, body-only and whole body. Tungsten bricks were used to shield either the head or the body, as required. The bricks were removed for whole-body exposures. The non-irradiated control rats (0 cGy) were taken to the NSRL, but not exposed. Nominal dose rates were between 1 and 10 cGy/min, depending upon the total dose.

**Behavior:** The animals were shipped to UMBC for behavioral testing. Cognitive performance was measured using the elevated-plus-maze (baseline anxiety); novel object recognition (general learning and memory); novel spatial location (spatial learning and memory); and operant responding on an ascending fixed-ratio schedule (motivation and responsiveness to environmental stimuli). The basis for the selection of the behavioral tests was that the behaviors are dopamine-mediated or show deterioration as a function of age.

**Results:** The results of the experiments indicate that: (i) there may be differences in cognitive performance following exposure to either head-only or whole-body exposure to NASA-relevant doses of ^16^O particles depending on the specific task; and (ii) for some tasks, body-only exposures can disrupt neurocognitive performance. The results also suggest that exposures involving the body may influence the responsiveness of the organism to the disruptive effects of exposure to HZE particles on neurocognitive performance. This effect is seen in two ways: first, as a direct effect of body-only exposure on cognitive performance, as shown by decreased time spent in a novel location compared with non-irradiated controls, suggesting impaired spatial location memory; and second, as an interaction between head and body exposures seen with whole-body exposures.

**Discussion:** These results indicate that body-only exposures have the potential to influence cognitive performance which is mediated by the brain. Although the mechanism remains to be fully established, it is possible that exposure of the body to HZE particles causes the release of cytokines which can affect neuronal function, either directly or through the mediation of the vagus nerve. As such, the results of studies using head-only or whole-body exposures may not be comparable. Also, astronauts may be subject to increased risk of deficits in cognitive performance during exploratory class missions.

